# A statistical package for evaluation of hybrid performance in plant breeding via genomic selection

**DOI:** 10.1038/s41598-023-39434-6

**Published:** 2023-07-27

**Authors:** Szu-Ping Chen, Chih-Wei Tung, Pei-Hsien Wang, Chen-Tuo Liao

**Affiliations:** Department of Agronomy, National Taiwan University, Taipei, Taiwan

**Keywords:** Plant sciences, Mathematics and computing

## Abstract

Hybrid breeding employs heterosis, which could potentially improve the yield and quality of a crop. Genomic selection (GS) is a promising approach for the selection of quantitative traits in plant breeding. The main objectives of this study are to (i) propose a GS-based approach to identify potential parental lines and superior hybrid combinations from a breeding population, which is composed of hybrids produced by a half diallel mating design; (ii) develop a software package for users to carry out the proposed approach. An R package, designated EHPGS, was generated to facilitate the employment of the genomic best linear unbiased model considering additive plus dominance marker effects for the hybrid performance evaluation. The R package contains a Bayesian statistical algorithm for calculating genomic estimated breeding value (GEBVs), GEBV-based specific combining ability, general combining ability, mid-parent heterosis, and better-parent heterosis. Three datasets that have been published in literature, including pumpkin (*Cucurbita maxima*), maize (*Zea mays*), and wheat (*Triticum aestivum* L.), were reanalyzed to illustrate the use of EHPGS.

## Introduction

Hybrid plant breeding can potentially be used as a method that employs heterosis to boost yield stability, allow the combination of dominant major genes, and offer a built-in plant variety protection system^1^ https://www.pnas.org/doi/full/10.1073/pnas.1514547112. Several field, vegetable, and flower crops use hybrids, including maize, sorghum, and sunflower. Interestingly, hybrid rice has been adopted and hybrid wheat research is drawing new attention^2^. Therefore, it is important and challenging to develop a highly efficient approach for identifying potential parental lines and superior hybrid combinations from many possible candidates. To create such an approach, we constructed a prediction model to screen out the desired individuals based on genomic selection (GS)^3^. To facilitate practical applications, we also generated a software package to implement our proposed GS-based approach.

Diallel mating designs have been traditionally used to evaluate the combining ability of parental lines in hybrids and to predict hybrid performance on quantitative traits of interest. To analyze diallel crosses, the total genetic variability is often separated into the general combining ability (GCA) for parental lines, and the specific combining ability (SCA) for hybrid combinations. The GCA is a measure of additive gene activity that relates to the average performance of a particular inbred line in hybrid combinations. The SCA is a measure of combining ability that links to the non-additive effects, including dominance and epistatic effects. In addition, mid-parent heterosis (MPH) is defined as the difference between a hybrid’s performance and the average performance of its parental lines, while better-parent heterosis (BPH) is defined as hybrid performance superior to the higher or better parental line^4^. However, the number of crossing combinations can be prohibitively high for extensive testing in a field experiment.

Due to the availability of high-density single nucleotide polymorphism (SNP) markers across an entire genome, GS becomes a promising approach to reduce cost and accelerate breeding cycles for plant breeding^5,6^. The conceptual basis of GS is the utilization of a training population with known phenotype and genotype data to build a prediction model that uses individuals with known genotype data only to predict genomic estimated breeding values (GEBVs)^7^. This GS-based approach has been applied to predict hybrid performance for several crops, such as barley^8^, maize^9,10^, rice^11,12^, wheat^13,14^, and pumpkin^15^. More recently, hybrid rice performance based on parental characteristics was evaluated using artificial neural networks, adaptive neuro-fuzzy inference system, and support vector machine^16^.

In this study, we obtained the required estimates for hybrid performance evaluation based on a GBLUP model, which took both additive and dominance marker effects into account. The GBLUP model was built based on a training population with known phenotype and genotype data. Here, we proposed a Bayesian statistical algorithm for the parameter estimation. Three datasets that have been published in literature, including pumpkin (*Cucurbita maxima*), maize (*Zea mays*), and wheat (*Triticum aestivum* L.), were reanalyzed to illustrate the application of our proposed approach.

## Materials and methods

### The genomic selection-based approach

#### The GBLUP model

The GBLUP model considering additive plus dominance effects can be described as follows:

1 $${\varvec{y}}={1}_{n}\mu +{{\varvec{g}}}_{A}+{{\varvec{g}}}_{D}+{\varvec{e}},$$

where ${\varvec{y}}$ is the vector of the phenotypic values; ${1}_{n}$ is the unit vector of length *n* (here *n* is the number of phenotypic values); ${{\varvec{g}}}_{A}$ is the vector of genotypic values for the additive effects; ${{\varvec{g}}}_{D}$ is the vector of genotypic values for the dominance effects; and ${\varvec{e}}$ is the vector of random errors. It is assumed that ${{\varvec{g}}}_{A}$, ${{\varvec{g}}}_{D}$, and ${\varvec{e}}$ are mutually independent and follow multivariate normal distributions, denoted by ${{\varvec{g}}}_{A}\sim N\left(0, {{\sigma }_{A}^{2}{\varvec{K}}}_{A}\right)$, ${{\varvec{g}}}_{D}\sim N\left(0, {{\sigma }_{D}^{2}{\varvec{K}}}_{D}\right)$, and ${\varvec{e}}\sim N\left(0, {\sigma }_{e}^{2}{{\varvec{I}}}_{n}\right)$. Here, ${{\varvec{K}}}_{A}=\frac{1}{p}{({\varvec{X}}}_{A}{{\varvec{X}}}_{A}^{T})$ is the genomic relationship matrix for the additive effects, abbreviated as A-GRM; the variance component ${\sigma }_{A}^{2}$ represents the cumulative variability of additive marker effects, abbreviated as A-VC; ${{\varvec{K}}}_{D}=\frac{1}{p}{({\varvec{X}}}_{D}{{\varvec{X}}}_{D}^{T})$ is the genomic relationship matrix for the dominance effects, abbreviated as D-GRM; and the variance component ${\sigma }_{D}^{2}$ represents the cumulative variability of the dominance marker effects, abbreviated as D-VC. For the additive effects, the SNP at each locus is coded as − 1, 0, or 1 for the homozygote of the minor allele, the heterozygote, and the homozygote of the major allele, respectively. For the dominance effects, the marker score is coded as 1 for the heterozygote, and 0 for both homozygotes. Then, ${{\varvec{X}}}_{A}$ and ${{\varvec{X}}}_{D}$ are the standardized marker score matrices for the additive effects and dominance effects, respectively, and $p$ is the number of the SNP markers.

### Estimation for GEBVs and genomic heritability

Let $\widehat{\mu }$ be the best linear unbiased estimate (BLUE) for $\mu$, ${\widehat{{\varvec{g}}}}_{A}$ be the BLUP for ${{\varvec{g}}}_{A}$, and ${\widehat{{\varvec{g}}}}_{D}$ be the BLUP for ${{\varvec{g}}}_{D}.$ Then, $\widehat{\mu }$, ${\widehat{{\varvec{g}}}}_{A}$, and ${\widehat{{\varvec{g}}}}_{D}$ can be obtained from the Henderson’s equations^17^:

2 $$\left[\begin{array}{ccc}n& {1}_{n}^{T}& {1}_{n}^{T}\\ {1}_{{\varvec{n}}}& {{\varvec{I}}}_{n}+{{{\varvec{K}}}_{A}^{-1}\lambda }_{A}& {{\varvec{I}}}_{n}\\ {1}_{{\varvec{n}}}& {{\varvec{I}}}_{n}& {{\varvec{I}}}_{n}+{{{\varvec{K}}}_{D}^{-1}\lambda }_{D}\end{array}\right]\left[\begin{array}{c}\widehat{\mu }\\ \begin{array}{c}{\widehat{{\varvec{g}}}}_{A}\\ {\widehat{{\varvec{g}}}}_{D}\end{array}\end{array}\right]=\left[\begin{array}{c}{1}_{n}^{T}y\\ y\\ y\end{array}\right],$$

where ${\lambda }_{A}={\sigma }_{e}^{2}/{\sigma }_{A}^{2}$ and ${\lambda }_{D}={\sigma }_{e}^{2}/{\sigma }_{D}^{2}$. Here, ${\lambda }_{A}$ and ${\lambda }_{D}$ can be replaced with suitable estimates for ${\sigma }_{e}^{2}$, ${\sigma }_{A}^{2}$, and ${\sigma }_{D}^{2}$, respectively denoted by ${\widehat{\sigma }}_{e}^{2}$, ${\widehat{\sigma }}_{A}^{2}$, and ${\widehat{\sigma }}_{D}^{2}$. The estimate for genomic heritability was then obtained as:

3 $${h}^{2}=\frac{{\widehat{\sigma }}_{A}^{2}+{\widehat{\sigma }}_{D}^{2}}{{\widehat{\sigma }}_{A}^{2}+{\widehat{\sigma }}_{D}^{2}+{\widehat{\sigma }}_{e}^{2}}.$$

In this study, the breeding population was composed of all possible hybrid combinations in a half diallel mating design. Let ${{\varvec{K}}}_{A}^{(bp)}$ and ${{\varvec{K}}}_{D}^{(bp)}$ respectively denote the A-GRM and D-GRM between the breeding population and the training population. Moreover, let ${\widehat{{\varvec{g}}}}_{A}^{(bp)}$ and ${\widehat{{\varvec{g}}}}_{D}^{(bp)}$ denote the BLUPs for the breeding population of additive and dominance effects, respectively. From the article^18^, ${\widehat{{\varvec{g}}}}_{A}^{(bp)}$ and ${\widehat{{\varvec{g}}}}_{D}^{(bp)}$ can be obtained as:

4 $${\widehat{{\varvec{g}}}}_{A}^{(bp)}={{\varvec{K}}}_{A}^{(bp)}{{\varvec{K}}}_{A}^{-1}{\widehat{{\varvec{g}}}}_{A},$$

and

5 $${\widehat{{\varvec{g}}}}_{D}^{(bp)}={{\varvec{K}}}_{D}^{(bp)}{{\varvec{K}}}_{D}^{-1}{\widehat{{\varvec{g}}}}_{D}.$$

The genomic estimated genotypic values for the individuals in the breeding population were then predicted by:

6 $${\widehat{{\varvec{y}}}}^{(bp)}={1}_{{N}_{1}}\widehat{\mu }+{\widehat{{\varvec{g}}}}_{A}^{(bp)}+{\widehat{{\varvec{g}}}}_{D}^{(bp)},$$

where ${N}_{1}$ is the number of hybrid combinations in the breeding population. Here, ${N}_{1}={C}_{2}^{{N}_{0}}$ with ${N}_{0}$ as the number of parental lines.

### Estimation for GCA, SCA, MPH, and BPH

Let ${GCA}_{i}$ and ${GCA}_{j}$ separately denote the GCAs for the parental lines ${P}_{i}$ and ${P}_{j}$, and ${SCA}_{ij}$ denote the SCA for their hybrid combination *P*_*i*_ ⨂ *P*_*j*_. Moreover, let ${g}_{A}^{(ij)}$ and ${g}_{D}^{(ij)}$ denote the BLUPs for *P*_*i*_ ⨂ *P*_*j*_ of additive and dominance effects, respectively. From the article^19^,

7 $${g}_{A}^{(ij)}={GCA}_{i}+{GCA}_{j},$$

and

8 $${g}_{D}^{(ij)}={SCA}_{ij}.$$

From Eq. (8), the BLUP for ${SCA}_{ij}$ was obtained as:

9 $${\widehat{SCA}}_{ij}={\widehat{g}}_{D}^{(ij)}.$$

Let

10 $$ \overline{G}_{A}^{\left( i \right)} = \frac{{\mathop \sum \nolimits_{j \ne i}^{{N_{0} }} \hat{g}_{A}^{{\left( {ij} \right)}} }}{{N_{0} - 1}} $$

and

11 $${\overline{G} }_{A}=\frac{{\sum }_{i=1}^{{N}_{0}}{\sum }_{j\ne i}^{{N}_{0}}{\widehat{g}}_{A}^{(ij)}}{{N}_{1}}$$

where ${\overline{G} }_{A}^{(i)}$ is the average over the additive genotypic values of the parental line *i*, and ${\overline{G} }_{A}$ is the average over all of the additive genotypic values. From Eq. (7), the BLUP for ${GCA}_{i}$ is given by:

12 $${\widehat{GCA}}_{i}=\frac{{(N}_{0}-1){\overline{G} }_{A}^{(i)}}{{N}_{0}-2}-\frac{{N}_{0}{\overline{G} }_{A}}{2({N}_{0}-2)}.$$

From the article^15^, the GEBV-based MPH and BPH for *P*_*i*_ ⨂ *P*_*j*_ can be estimated by:

13 $${\widehat{MPH}}_{ij}={\widehat{SCA}}_{ij}$$

and

14 $${\widehat{BPH}}_{ij}={\widehat{SCA}}_{ij}-\left|{\widehat{GCA}}_{i}-{\widehat{GCA}}_{j}\right|$$

where $\left|{\widehat{GCA}}_{i}-{\widehat{GCA}}_{j}\right|$ is the absolute value of (${\widehat{GCA}}_{i}-{\widehat{GCA}}_{j}$). Under the positive heterosis assumption, the value of MPH or BPH is larger, and the heterosis of the hybrid combination is stronger.

### The Bayesian statistical algorithm

For a given training population with known phenotype and genotype data, a Bayesian Gibbs sampling (BGS) algorithm, modified from an algorithm presented in the article^20^, was used to estimate the required parameters. The algorithm can be described as follows.

- Step 1: Set initial values for the parameters in the model.

The default values are given by:

$\upmu =\overline{y }$ (the sample mean of the phenotypic values), ${{\varvec{g}}}_{A}={{\varvec{g}}}_{D}=0$, ${\sigma }_{e}^{2}=1$, and ${\sigma }_{A}^{2}={\sigma }_{D}^{2}=0.5$.

- Step 2: Rewrite Eq. (2) as
  15 $$\left[\begin{array}{ccc}{{\varvec{C}}}_{11}& {{\varvec{C}}}_{12}& {{\varvec{C}}}_{13}\\ {{\varvec{C}}}_{21}& {{\varvec{C}}}_{22}& {{\varvec{C}}}_{23}\\ {{\varvec{C}}}_{31}& {{\varvec{C}}}_{32}& {{\varvec{C}}}_{33}\end{array}\right]\left[\begin{array}{c}{{\varvec{g}}}_{1}\\ {{\varvec{g}}}_{2}\\ {{\varvec{g}}}_{3}\end{array}\right]=\left[\begin{array}{c}{{\varvec{\gamma}}}_{1}\\ {{\varvec{\gamma}}}_{2}\\ {{\varvec{\gamma}}}_{3}\end{array}\right].$$

Update ${{\varvec{g}}}_{i}$ by ${{\varvec{g}}}_{i}\sim N({{\varvec{g}}}_{i}^{*}, {\sigma }_{e}^{2}{{\varvec{C}}}_{ii}^{-1})$, where ${{\varvec{g}}}_{i}^{*}={{\varvec{C}}}_{ii}^{-1}({{\varvec{\gamma}}}_{i}-{{\varvec{C}}}_{i,-i}{{\varvec{g}}}_{-i})$ for *i* = 1, 2, 3. Here, ${{\varvec{C}}}_{i,-i}$ denotes ${{\varvec{C}}}_{i,j}$ for all $j\ne i$; and ${{\varvec{g}}}_{-i}$ is ${{\varvec{g}}}_{j}$ for all $j\ne i$.

- Step 3: Calculate the vector of residuals as: ${\varvec{e}}={\varvec{y}}-{{\varvec{g}}}_{1}-{{\varvec{g}}}_{2}-{{\varvec{g}}}_{3}$.
- Step 4: Update ${\sigma }_{e}^{2}$ as ${\sigma }_{e}^{2}=({{\varvec{e}}}^{T}{\varvec{e}}+{S}^{\boldsymbol{*}}{v}^{\boldsymbol{*}})/{\chi }_{n+{v}^{\boldsymbol{*}}}^{2}$, where ${\chi }_{n+{v}^{\boldsymbol{*}}}^{2}$ is the chi-square random variate with $n+{v}^{\boldsymbol{*}}$ degrees of freedom; ${S}^{\boldsymbol{*}}=0.5V$ with* V* as the sample variance of the values in ${\varvec{y}}$; and ${v}^{\boldsymbol{*}}=5$.
- Step 5: Update ${\sigma }_{A}^{2}$ as ${\sigma }_{A}^{2}=({{\varvec{g}}}_{A}^{T}{{\varvec{K}}}_{A}^{-1}{{\varvec{g}}}_{A}+{S}^{\boldsymbol{*}}{v}^{\boldsymbol{*}})/{\chi }_{n+{v}^{\boldsymbol{*}}}^{2}$; and ${\sigma }_{D}^{2}$ as ${\sigma }_{D}^{2}=({{\varvec{g}}}_{D}^{T}{{\varvec{K}}}_{D}^{-1}{{\varvec{g}}}_{D}+{S}^{\boldsymbol{*}}{v}^{\boldsymbol{*}})/{\chi }_{n+{v}^{\boldsymbol{*}}}^{2}$.
- Step 6: Update the equations in Eq. (15) with ${\lambda }_{A}={\sigma }_{e}^{2}/{\sigma }_{A}^{2}$, and ${\lambda }_{D}={\sigma }_{e}^{2}/{\sigma }_{D}^{2}$.
- Step 7: Repeat Steps 2–6 K times to generate a series of results over the K iterations, which are denoted by:
- $${\mu }^{(k)}$$, $${{\varvec{g}}}_{A}^{(k)}$$, $${{\varvec{g}}}_{D}^{(k)}$$, $${\sigma }_{A}^{2(k)}$$, $${\sigma }_{D}^{2(k)}$$, and $${\sigma }_{e}^{2(k)}$$ for $$k=1, 2, \cdots , \mathrm{K}$$.
- Step 8: Discard the results from the first $0.9\mathrm{K}$ iterations, and average the results from the remaining 0.1 K iterations. The number of iterations K is defaulted as 5000.
- Step 9: Repeat Steps 1–8 M times to generate M sets of the averages of the parameters generated from Step 8. The number of chains M is defaulted as five.
- Step 10: Average the resulting mean values of the parameters over the M chains, and the resulting averages are treated as the estimates for the parameters.

An R package called as EHPGS generated for executing the proposed approach is available from GitHub (https://github.com/spcspin/EHPGS). A referenced manual and a tutorial including a demonstration example are provided in the package.

### A comparison study

The pumpkin dataset was analyzed using a two-stage approach in the article^15^, in which the authors first estimated GEBVs, SCAs, GCAs, MPHs, and BPHs based on a whole genome regression model using Bayes C estimation in the R package BGLR^21^. Then, they calculated A-GRM and D-GRM by the two different formulas^22,23^. The restricted maximum likelihood estimation (REML) method was performed for estimating the variance components by using another R package sommer^24^. A comparison of the results obtained from the two-stage approach and ours was discussed in the next section.

The Bayesian reproducing kernel Hilbert space (RKHS) method in BGLR is another Bayesian algorithm that has been commonly used to perform GEBV prediction for the GBLUP model in Eq. (1). To compare the use of the Bayesian RKHS method with our proposed BGS algorithm, the three datasets was reanalyzed by using BGLR. The priors specified in BGLR were the same as ours, the number of iterations was set to 10,000, the number of burn-in was fixed at 9000, and the number of chains was set to five (the BGLR function was repeatedly run five times). These settings are exactly the same as our algorithm in analyzing the datasets.

### A simulation study

To further examine whether the proposed BGS algorithm can more accurately estimate known variance components compared to established methods, such as the REML method in sommer, and the Bayesian RKHS method in BGLR, a simulation study was conducted as follows. The estimated values for the model parameters obtained from the training data (displayed in Table 3) were used to generate 3000 sets of phenotype data for the training population in each dataset (119, 276, and 600 realized observations in each simulated dataset for the pumpkin, maize, and wheat datasets, respectively). For a stimulated dataset, the variance components were estimated by the REML, Bayesian RKHS, and our BGS methods.

### A cross-validation analysis

A tenfold cross-validation analysis using empirical data was also performed to compare the accuracy on GEBV prediction among the three methods. There were 119 and 276 empirical observations available in the pumpkin and maize datasets, respectively. For the sake of computational cost saving, 500 individuals randomly selected from the 2556 available hybrids in the wheat dataset were used for this analysis. The procedure can be described as follows. Step 1: Each of the three datasets was partitioned into 10 exclusive clusters at random. Step 2: During the cross-validation process, each of the 10 clusters was progressively and alternately used as the testing set. At the same time, the remaining nine clusters were pooled as the training set. Step 3: After the GEBV prediction by each method, Pearson’s correlation between GEBVs and phenotypic values in the testing set was calculated for each dataset. Here, the procedure was repeated five times to generate 50 correlation coefficients for each dataset.

### The genome datasets

Three datasets that have been published in literature were reanalyzed to illustrate the use of EHPGS.

#### Pumpkin dataset

A pumpkin dataset which contained 119 intra-crossing hybrid combinations of *C. maxima* with phenotypic values for fruit weight (FWT) (kg) was analyzed for evaluation of hybrid performance^15^. The phenotype data were historical data collected from 1988 to 2016. All the trials were conducted at a single location experiment in southern area of Taiwan. Every hybrid had six to ten observations at each time point, and the average of them was used as the phenotypic observation for the hybrid of the year. Because the phenotypic values of every hybrid were observed for more than one year, the different year effects were therefore removed based on the assumption that they were random effects following a normal distribution.

The germplasm collection of the pumpkin set consisted of 320 parental lines, which were classified into three clusters: *C. maxima* with 142 inbred lines, *C. pepo* with 60 inbred lines and *C. moschata* with 118 inbred lines. After SNP calling, 76,815 SNPs were extracted from the parental lines, and only 4,521 SNPs remaining for *C. maxima* after the filtering by missing rate ≥ 0.05, minor allele frequency (MAF) < 0.05, and a series of operations for determining linkage disequilibrium (LD) blocks. The 142 inbred lines produced ${C}_{2}^{142}=\mathrm{10,011}$ potential hybrid combinations in a half diallel mating design. The means adjusted from the year effects for the 119 *C. maxima* hybrids were used in the current study to build a GBLUP model for evaluating the performance of the 10,011 hybrid combinations.

#### Maize dataset

A maize dataset was analyzed to study the optimal designs for GS in hybrid crops, which consisted of 276 hybrids derived from 24 parental lines in a half diallel mating design^2^. The 24 diverse parents were classified into two groups according to the germplasm origin and a principal component analysis. The two groups were (i) the temperate and mixed (TM) group, consisting of 11 inbred lines (i.e., B73, B97, Ky21, M162W, Mo17, MS71, Oh43, OH7B, M37W, Mo18W, and Tx303); and (ii) the tropical and sub-tropical (TS) group consisting of the remaining 13 inbred lines (i.e., CML52, CML69, CML103, CML228, CML247, CML277, CML322, CML333, Ki3, Ki11, NC350, NC358, and Tzi8). There were ${C}_{2}^{11}=55$ hybrid combinations in the TM group, ${C}_{2}^{13}=78$ hybrids in the TS group, and 11 × 13 = 143 hybrids between the two groups. Three trait values, flowering time, ear height, and grain yield (YLD) (Mg/ha), were evaluated for all of the hybrids at two locations (i.e., Columbia, MO and Clayton, NC) in 2005 and 2006. In our study, the combined BLUP values from the two locations for YLD were evaluated.

Genotype data for the 24 inbred lines were extracted from the Maize HapMap V2^25^ at www.panzea.org, which consisted of 10,296,310 SNP markers. The SNP markers were first filtered by missing rate ≥ 0.05 and MAF ≤ 0.1, resulting in 134,726 SNPs remaining. Missing genotypes were then imputed with the homozygote of the major allele. To screen out reliable SNPs for building a GBLUP model, the retained SNPs were further filtered by LD blocks. The LD parameter ${r}^{2}$ (i.e., the squared Pearson’s correlation coefficient) of the SNPs for each chromosome was estimated using TASSEL5.2.41^26^ with a sliding window = 10. A smooth function between ${r}^{2}$ and the physical distance (bp) was built using an R function *loess.smooth*( ) with a second-degree locally weighted polynomial regression. The LD decay of ten chromosomes is displayed in Fig. S1 of the Supplementary Materials. Filtering the 134,726 SNP markers by the LD block sizes if ${r}^{2}$ approached 0.2, resulting in 46,134 SNPs remaining. A SNP was also deleted if its corresponding column for the dominance effects was a zero vector. Finally, 30,239 SNP markers were retained for further analysis. In the current study, all 276 hybrids with known trait values were used as the training population for the prediction model construction.

#### Wheat dataset

A genome-based establishment of a high-yielding heterotic pattern for hybrid wheat breeding was investigated, and the study was based on 135 advanced elite winter wheat lines^27^. A set of 1604 wheat hybrids produced from crosses among the 15 male lines and 120 female lines were then evaluated for grain yield (YLD) (Mg/ha) in 11 environments. Grain yield data for all ${C}_{2}^{135}=9045$ unique hybrids were predicted based on those of the phenotyped individuals. For the genotype data, the 135 lines were fingerprinted by using a 90,000 SNP array based on an Illumina Infinium array. After quality tests, 17,372 high-quality SNP markers were retained.

To study optimal designs for GS, 2556 hybrid combinations, produced by the half diallel mating design on 72 lines selected from the original 135 elite wheat lines, were analyzed in the article^2^. An optimal training population with 600 individuals, determined by the r-score criterion^28^, was used in the current study to build the GBLUP model for the performance evaluation on the 2556 hybrid combinations.

## Results and Discussion

### Pumpkin dataset

By the half diallel mating design, the 142 parental lines produced ${C}_{2}^{142}=\mathrm{10,011}$ hybrid combinations in the breeding population. For illustration purposes, we only reported the top 25 superior hybrid combinations with the largest GEBVs, together with their SCAs, MPHs, and BPHs in Table 1; and the top 10 potential parental lines with the largest GCAs in Table 2. Table 1 illustrates the important finding that both ${MPH}_{ij}$ and ${BPH}_{ij}$ are greater than 0 for all of the selected hybrids, showing that they had better performance in FWT than both of their parents. More interestingly, every superior hybrid presented in Table 1 was derived from one or two of the potential parental lines presented in Table 2. Particularly, P026, the parental line with the highest GCA, involved the top 11 hybrids with the greatest GEBVs among the 25 selected hybrids.

**Table 1:** The top 25 superior hybrid combinations with the largest GEBVs for fruit weight (FWT) within a pumpkin population.

*P*_*i*_ ⨂ *P*_*j*_	${GEBV}_{ij}$	${SCA}_{ij}$	${MPH}_{ij}$	${BPH}_{ij}$
P026 ⨂ P236	3.432	0.686	0.686	0.648
P026 ⨂ P234	3.396	0.676	0.676	0.612
P026 ⨂ P235	3.385	0.660	0.660	0.601
P026 ⨂ P027	3.362	0.656	0.656	0.578
P026 ⨂ P028	3.321	0.626	0.626	0.537
P026 ⨂ P237	3.315	0.646	0.646	0.531
P026 ⨂ P302	3.107	0.527	0.527	0.322
P007 ⨂ P026	3.105	0.526	0.526	0.321
P026 ⨂ P254	3.062	0.440	0.440	0.277
P026 ⨂ P253	3.060	0.433	0.433	0.276
P026 ⨂ P255	3.034	0.425	0.425	0.250
P227 ⨂ P236	3.012	0.569	0.569	0.302
P227 ⨂ P235	3.005	0.584	0.584	0.337
P227 ⨂ P234	2.999	0.582	0.582	0.341
P100 ⨂ P234	2.998	0.532	0.532	0.340
P100 ⨂ P235	2.990	0.520	0.520	0.323
P100 ⨂ P236	2.982	0.490	0.490	0.272
P026 ⨂ P252	2.974	0.356	0.356	0.189
P234 ⨂ P313	2.959	0.619	0.619	0.300
P235 ⨂ P313	2.950	0.605	0.605	0.281
P027 ⨂ P100	2.947	0.495	0.495	0.318
P028 ⨂ P227	2.936	0.544	0.544	0.328
P236 ⨂ P313	2.930	0.564	0.564	0.219
P028 ⨂ P100	2.926	0.485	0.485	0.318
P027 ⨂ P227	2.923	0.521	0.521	0.293

Note that ${GEBV}_{ij}$ is the genomic estimated breeding value; ${SCA}_{ij}$ is the specific combining ability; ${MPH}_{ij}$ is the mid-parent heterosis; ${BPH}_{ij}$ is the better-parent heterosis for hybrid *P*_*i*_ ⨂ *P*_*j*_*.*

**Table 2:** The top 10 potential parental lines with the largest GCAs for fruit weight (FWT) within a pumpkin population.

${P}_{i}$	${GCA}_{i}$
P026	0.6143
P236	0.5766
P235	0.5552
P234	0.5506
P027	0.5360
P028	0.5251
P237	0.4993
P253	0.4565
P254	0.4514
P252	0.4473

${GCA}_{i}$ is the general combining ability for parental line ${P}_{i}$.

The estimates for the variance components and genomic heritability are shown in Table 3*.* From the table, the estimates of the A-VC, D-VC, and genomic heritability are given by ${\widehat{\sigma }}_{A}^{2}=$ 0.306, ${\widehat{\sigma }}_{D}^{2}=0.159$, and ${h}^{2}=0.807$. The high heritability explains why the values of ${MPH}_{ij}$ and ${BPH}_{ij}$ in Table 1 are all positive, and indicates strong heterosis in FWT among the intra-crossing hybrid combinations of *C. maxima*.

**Table 3:** The estimates for the variance components, genomic heritability, and constant term in fruit weight (FWT) for a pumpkin dataset and in yield (YLD) for maize, and wheat datasets.

Dataset	${\widehat{\sigma }}_{A}^{2}$	${\widehat{\sigma }}_{D}^{2}$	${\widehat{\sigma }}_{e}^{2}$	${h}^{2}$	$\widehat{\mu }$
Pumpkin	0.306	0.159	0.111	0.807	1.577
Maize	0.434	0.420	1.202	0.415	11.567
Wheat	0.066	0.014	0.002	0.976	10.792

### Maize dataset

There were ${C}_{2}^{24}=276$ hybrid combinations derived from the 24 parental lines.

For illustration purposes, we reported the top 15 superior hybrids with the largest GEBVs, together with their SCAs, MPHs, and BPHs in Table 4; and the top 5 potential parental lines with the largest GCAs in Table 5. From Table 4, both ${MPH}_{ij}$ and ${BPH}_{ij}$ are greater than 0 for all of the selected hybrids, showing that they had better performance in YLD than both of their parents. A total of 12 out of the 15 selected hybrids belong to the inter-crossing group between TM and TS. From Table 5, the top five parental lines with the greatest GCAs are CML228, CML103, Mo17, B97, and B73, and involved all of the 15 superior parental lines, with the exception of MS71 ⨂ Tzi8.

**Table 4:** The top 15 superior hybrid combinations with the largest GEBVs for grain yield (GYD) within a maize population.

*P*_*i*_ ⨂ *P*_*j*_	${GEBV}_{ij}$	${SCA}_{ij}$	${MPH}_{ij}$	${BPH}_{ij}$
OH7B ⨂ CML228*	12.975	0.587	0.587	0.385
B73 ⨂ CML228*	12.697	0.635	0.635	0.452
MO17 ⨂ TZI8*	12.589	0.582	0.582	0.454
MO18W ⨂ CML103*	12.512	0.447	0.447	0.228
M162W ⨂ CML228*	12.504	0.460	0.460	0.239
TX303 ⨂ CML228*	12.499	0.358	0.358	0.132
B73 ⨂ CML69*	12.476	0.347	0.347	0.308
OH43 ⨂ CML228*	12.463	0.389	0.389	0.182
MO17 ⨂ OH7B	12.460	0.466	0.466	0.400
MO17 ⨂ CML228*	12.450	0.401	0.401	0.265
B73 ⨂ M162W	12.432	0.336	0.336	0.298
CML52 ⨂ CML103	12.361	0.294	0.294	0.076
MS71 ⨂ TZI8*	12.360	0.474	0.474	0.462
MS71 ⨂ CML228*	12.331	0.342	0.342	0.065
B97 ⨂ CML103*	12.311	0.317	0.317	0.188

Note that ${GEBV}_{ij}$ is the genomic estimated breeding value; ${SCA}_{ij}$ is the specific combining ability; ${MPH}_{ij}$ is the mid-parent heterosis; ${BPH}_{ij}$ is the better-parent heterosis for hybrid *P*_*i*_ ⨂ *P*_*j*_*.*

*Represents a hybrid belonging to the inter-group between TM and TS.

**Table 5:** The top five potential parental lines with the largest GCAs for grain yield (GYD) within a maize population.

${P}_{i}$	${GCA}_{i}$
CML228^TS^	0.2361
CML103^TS^	0.1827
MO17™	0.0999
B97™	0.0538
B73™	0.0527

${GCA}_{i}$ is the general combining ability for parental line ${P}_{i}$.

*TS* the tropical and subtropical group, *TM* the temperate and mixed group.

The estimates for the variance components and genomic heritability are also displayed in Table 3. From the table, the estimates of the estimates of the A-VC, D-VC, and genomic heritability are given by ${\widehat{\sigma }}_{A}^{2}=$ 0.434, ${\widehat{\sigma }}_{D}^{2}=0.420$, and ${h}^{2}=0.415$, partially explaining why the values of ${MPH}_{ij}$ and ${BPH}_{ij}$ in Table 4 are all positive, and showing that there is an obvious heterosis in YLD within the breeding population.

### Wheat dataset

By the half diallel mating design, the 72 parental lines produced ${C}_{2}^{72}=2556$ hybrid combinations in the breeding population. For illustration purposes, we only reported the top 20 superior hybrids with the largest GEBVs, together with their SCAs, MPHs, and BPHs in Table 6; and the top 10 potential parental lines with the largest GCAs in Table 7. The estimates for the variance components and genomic heritability are also displayed in Table 3. From Table 6, all of the ${MPH}_{ij}$ are greater than 0, showing that they had a larger YLD than the mean YLD of their parents. Most of the ${BPH}_{ij}$ are noticeably smaller than ${MPH}_{ij}$, probably because the additive effects ${(\widehat{\sigma }}_{A}^{2}=$ 0.066, Table 3) were stronger than the dominance effects (${\widehat{\sigma }}_{D}^{2}=0.014$, Table 3). Moreover, 11 of the 20 BPHs are negative, showing that the corresponding hybrids were inferior to their better-parents. Every superior hybrid presented in Table 6 was derived from one or two of the potential parental lines presented in Table 7. Particularly, F102, the parental line with the highest GCA (Table 7), involved 17 of the 20 selected superior hybrids (Table 6).

**Table 6:** The top 20 superior hybrid combinations with the largest GEBVs for grain yield (GYD) within a wheat population.

*P*_*i*_ ⨂ *P*_*j*_	${GEBV}_{ij}$	${SCA}_{ij}$	${MPH}_{ij}$	${BPH}_{ij}$
F6 ⨂ F102	11.491	0.354	0.354	0.083
F102 ⨂ M6	11.436	0.070	0.070	0.032
F102 ⨂ M9	11.435	0.151	0.151	0.029
F97 ⨂ F102	11.414	0.191	0.191	0.007
F1 ⨂ F102	11.409	0.250	0.250	0.002
F46 ⨂ F102	11.401	0.344	0.344	− 0.008
F39 ⨂ F102	11.378	0.316	0.316	− 0.031
F20 ⨂ F102	11.370	0.141	0.141	− 0.037
F102 ⨂ xM14	11.361	0.090	0.090	− 0.045
F100 ⨂ F102	11.359	0.051	0.051	− 0.047
F97 ⨂ M6	11.355	0.169	0.169	0.024
F101 ⨂ F102	11.350	0.172	0.172	− 0.057
F44 ⨂ F102	11.342	0.200	0.200	− 0.066
F98 ⨂ F102	11.338	0.143	0.143	− 0.070
F99 ⨂ F102	11.337	0.221	0.221	− 0.072
F115 ⨂ M6	11.321	0.137	0.137	− 0.010
F61 ⨂ F97	11.320	0.315	0.315	0.277
F102 ⨂ F115	11.311	0.090	0.090	− 0.096
F102 ⨂ M1	11.295	0.150	0.150	− 0.113
F102 ⨂ M8	11.290	0.162	0.162	− 0.119

Note that ${GEBV}_{ij}$ is the genomic estimated breeding value; ${SCA}_{ij}$ is the specific combining ability; ${MPH}_{ij}$ is the mid-parent heterosis; ${BPH}_{ij}$ is the better-parent heterosis for hybrid *P*_*i*_ ⨂ *P*_*j*_*.*

**Table 7:** The top 10 potential parental lines with the largest GCAs for grain yield (GYD) within a wheat population.

${P}_{i}$	${GCA}_{i}$
F102	0.311
M6	0.272
F100	0.213
M9	0.188
M14	0.176
F20	0.133
F97	0.127
F3	0.126
F115	0.125
F84	0.122

${GCA}_{i}$ is the general combining ability for the parental line ${P}_{i}$.

In summary, the BPH values were consistently positive for the top hybrids in both the pumpkin and maize datasets, implying that there exists a strong and useful heterosis in the two crops. The valuable result can also be found in literature^15,29^. However, only a few of the top hybrids had a positive but too small BPH value in the case of wheat, indicating that the heterosis existing in this dataset may not be adequate for practical utility. A wheat hybrid has a small positive or negative BPH value because one of its parents is inferior^30^.

### The correlation between phenotypic values and GEBVs

**Figure 1:**
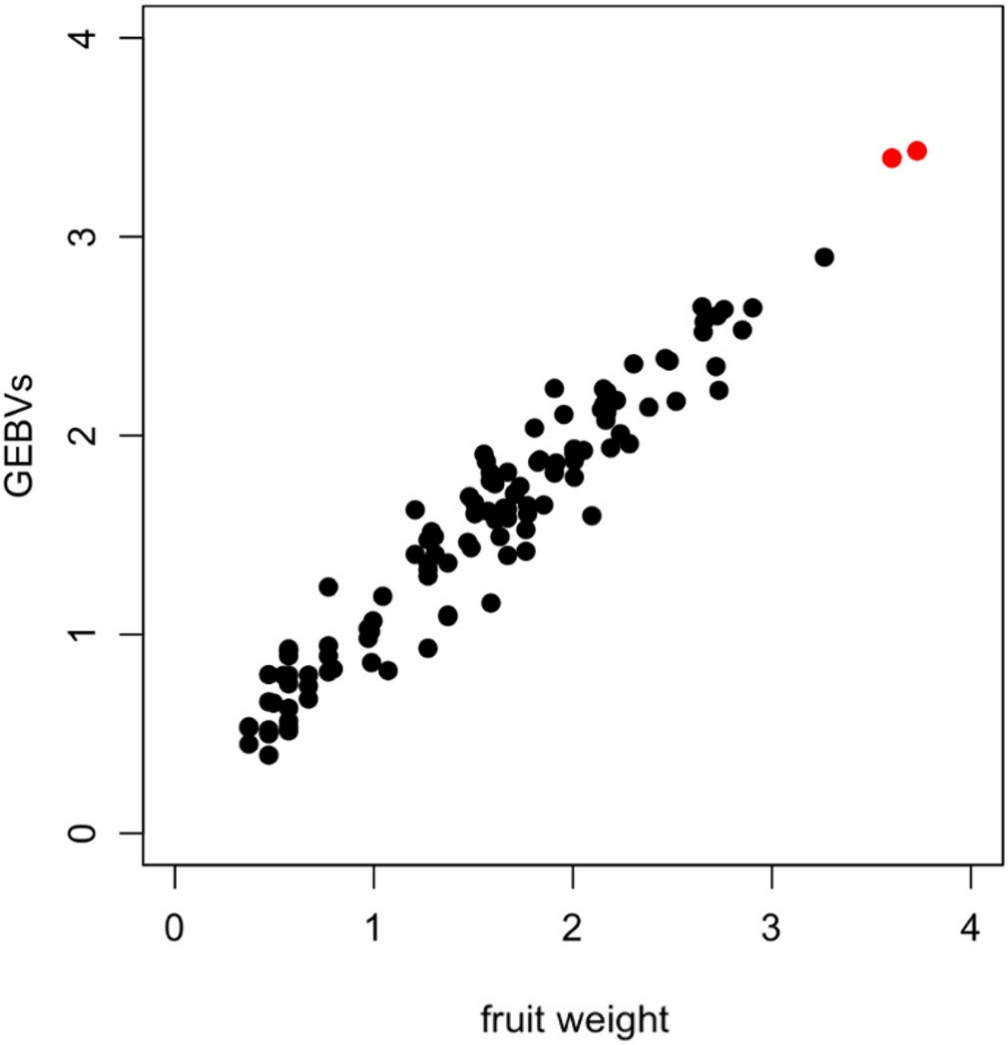
The scatter plot for all available phenotypic values (i.e., 119 individuals) and their GEBVs in the pumpkin dataset. The colored points represent the two hybrids out of the 25 selected superior hybrids. Note that the remaining 23 selected hybrids didn’t appear in the plot, because their phenotypic values were not available. Pearson’s correlation for these 119 points was calculated as $\mathrm{r}=0.9691$.

**Figure 2:**
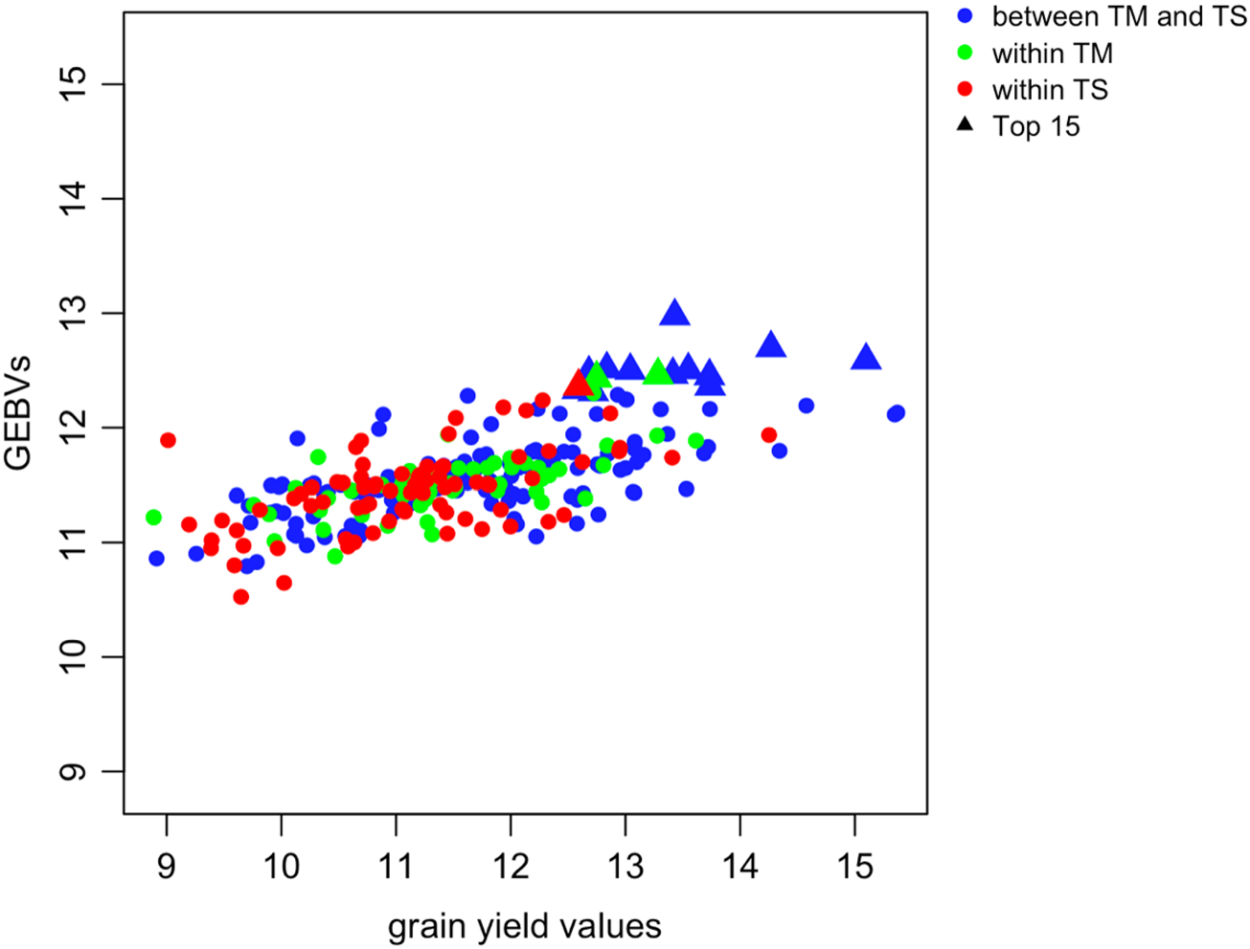
The scatter plot for all available phenotypic values (i.e., 276 individuals) and their GEBVs in the maize dataset. The triangle points represent the top 15 superior hybrid combinations with the highest GEBVs. Pearson’s correlation for these 276 points was calculated as $\mathrm{r}=0.6786$.

**Figure 3:**
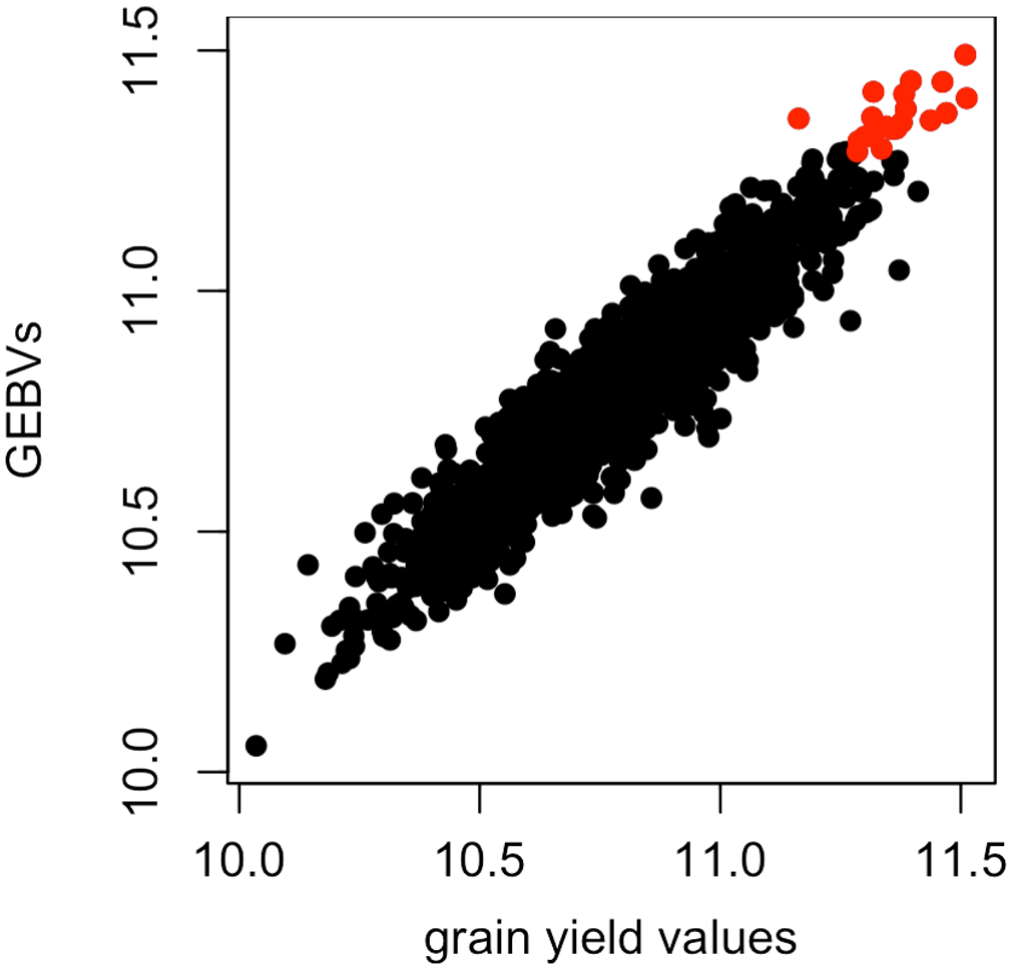
The scatter plot for all available phenotypic values (i.e., 2556 individuals) and their GEBVs in the wheat dataset. The colored points represent the top 20 superior hybrids with the highest GEBVs. Pearson’s correlation for these 2556 points was calculated as $\mathrm{r}=0.9445$.

Scatter plots of all available phenotypic values (119, 276, and 2556 individuals in the pumpkin, maize, and wheat datasets, respectively) and their GEBVs in each dataset are displayed in Figs. 1, 2 and 3. The respective Pearson’s correlation coefficients are 0.9691, 0.6786, and 0.9445. From the figures, most of the selected superior hybrids appeared in the upper right-hand corners, meaning that the selected hybrids with higher GEBVs also have higher actual phenotypic values. This is a valuable result because phenotypic selection is usually costly and time-consuming for selective breeding. The great consistency exists between the results of genomic selection and phenotypic selection, supporting that the proposed GS-based approach can be recommended for practical applications.

### The results of the comparison study

The top 25 superior hybrids identified by the two-stage approach^15^, together with those identified by our proposed approach are displayed in Table S1 of the Supplementary Materials. The corresponding identified 10 potential parental lines are displayed in Table S2. Both sets of the results are highly consistent with each other. From Table S1, 18 hybrids were in common among the 25 hybrids selected by each approach, and the top six hybrids with the highest GEBVs were the same, even though the order was slightly different. Table S2 indicates seven potential parental lines in common among the 10 selected by each approach. The variance components for additive, dominance, random error effects, and genomic heritability estimated are 0.195, 0.119, 0.066, and 0.826, respectively, from the two-stage approach. The corresponding estimates by our approach are 0.306, 0.159, 0.111 and 0.807. Even though the two corresponding estimates are different from each other, the two estimates of the genomic heritability are fairly close.

Overall, the results of the identified top parental lines and hybrid combinations between the Bayesian RKHS method in BGLR and our BGS algorithm were highly consistent with each other. Pearson’s correlations between GEBVs and phenotypic values for the datasets are displayed in Table 8. From which, our proposed algorithm led to higher Pearson’s correlations in the pumpkin and three maize datasets, but almost equal in the wheat dataset. Additionally, the estimates for variance components and genomic heritability by using the Bayesian RKHS method are displayed in Table 9. In comparison with those obtained from our BGS algorithm (Table 3), BGLR resulted in relatively low genomic heritability.

**Table 8:** Pearson’s correlations between GEBVs and phenotypic values for the datasets obtained from Bayesian RKHS method in BGLR and our proposed BGS algorithm.

Methods	Datasets				
	Pumpkin	Maize-A^1^	Maize-B^1^	Maize-C^1^	Wheat
BGLR	0.9461	0.6167	0.6580	0.6039	0.9439
Our algorithm	0.9691	0.6786	0.7560	0.6546	0.9445

^1^Maize-A: the combined data from the two locations; Maize-B: the data from Columbia, MO; Maize-C: the data from Clayton, NC.

**Table 9:** The estimates for the variance components and genomic heritability in fruit weight (FWT) for a pumpkin dataset and in yield (YLD) for maize, and wheat datasets by using Bayesian RKSH method in BGLR.

Dataset	${\widehat{\sigma }}_{A}^{2}$	${\widehat{\sigma }}_{D}^{2}$	${\widehat{\sigma }}_{e}^{2}$	${h}^{2}$
Pumpkin	0.156	0.144	0.132	0.694
Maize	0.042	0.284	1.253	0.207
Wheat	0.029	0.014	0.003	0.935

### The results of the simulation study and the cross-validation analysis

**Figure 4:**
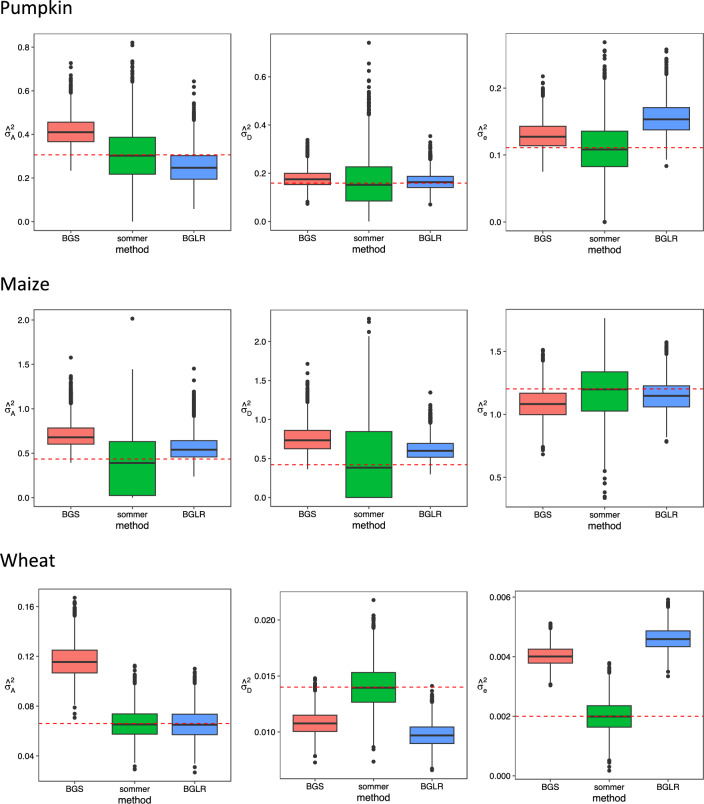
Side-by-side box-plots for the estimates of variance components over the 3000 simulated datasets by using the three different methods. The known values of the variance components in the simulation study are indicated as a red dashed line.

Side-by-side box-plots for the estimates of the variance components over the 3000 repetitions in the simulation study are displayed in Fig. 4. From the figure, the two Bayesian methods of BGS algorithm and the Bayesian RKHS method generally led to larger bias but smaller dispersion than the REML method in the estimation. The performance of the methods might be dependent on different dataset-variance-component combinations. For example, BGS algorithm tended to overestimate ${\sigma }_{A}^{2}$, but the Bayesian RKHS method was likely to underestimate it in the pumpkin dataset. Moreover, BGS algorithm had slightly better performance in ${\widehat{\sigma }}_{e}^{2}$, but worse in ${\widehat{\sigma }}_{D}^{2}$ than the Bayesian RKHS method in the dataset.

The mean and the standard deviation over the 50 resulting values in the cross-validation analysis are displayed in Table 10. From the table, the three methods had quite close performance in the three datasets. BGS algorithm, the REML method, and the Bayesian RKHS method outperformed the others in the maize, wheat, and pumpkin datasets, respectively. However, the margins were very small. According to the above results, the REML method in sommer and the Bayesian RKHS method in BGLR were also imported in EHPGS as options for the GEBV prediction and variance component estimation.

**Table 10:** Means and standard deviations (in parentheses) over the 50 resulting Pearson’s correlation coefficients in the cross-validation analysis.

Datasets	Methods		
	BGS	Sommer	BGLR
Pumpkin	0.7627	0.7522	0.7705
	(0.1403)	(0.1443)	(0.1367)
Maize	0.0996	0.0975	0.0977
	(0.1963)	(0.2015)	(0.1963)
Wheat	0.9267	0.9329	0.9266
	(0.0204)	(0.0186)	(0.0205)

## Conclusion

In this study, a software package called EHPGS was generated for identifying potential parental lines and superior hybrid combinations from a breeding population, which is composed of all possible hybrids produced by a half diallel mating design. A training population with known phenotype and genotype data is required to build the GBLUP model, and then a set of parental lines with known genotype data is also required to perform GEBV prediction for its derived hybrid combinations. Any dataset with such training population and parental line set can fit the package. For an input dataset, EHPGS generates GEBVs, SCAs, GCAs, MPHs, and BPHs for all potential candidates to achieve the task.

## Supplementary Information

Supplementary Information.

## Data Availability

All phenotype and genotype datasets that were analyzed in this study can be downloaded from Figshare (https://doi.org/10.6084/m9.figshare.22359883.v2).
