# Supplementary material for: A statistical package for evaluation of hybrid performance in plant breeding via genomic selection
Source: Sci Rep. 2023 Jul 27;13:12204. doi: 10.1038/s41598-023-39434-6 (PMC10374541; doi:10.1038/s41598-023-39434-6)
Supplement: Supplementary file 1 — Supplementary Information. [file 41598_2023_39434_MOESM1_ESM.docx]

**A Statistical Package for Evaluation of Hybrid Performance in Plant Breeding via Genomic Selection**

**Supplementary Materials**

Chromosome 4


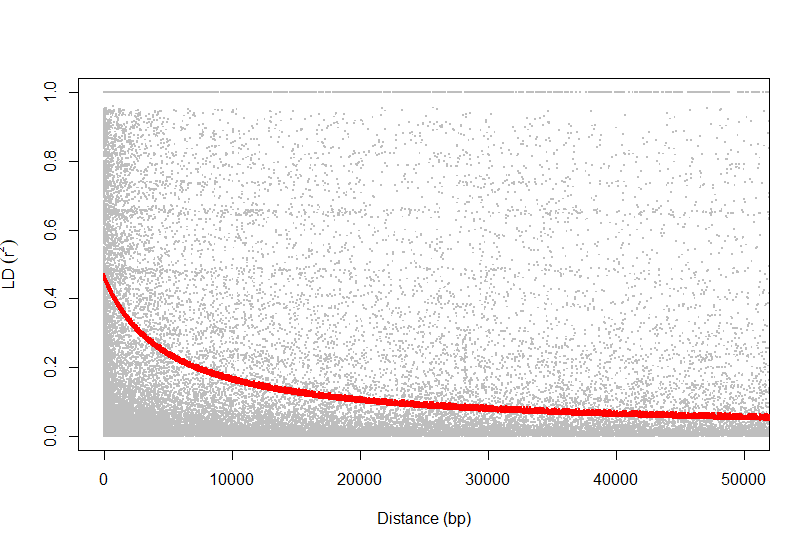


Chromosome 3


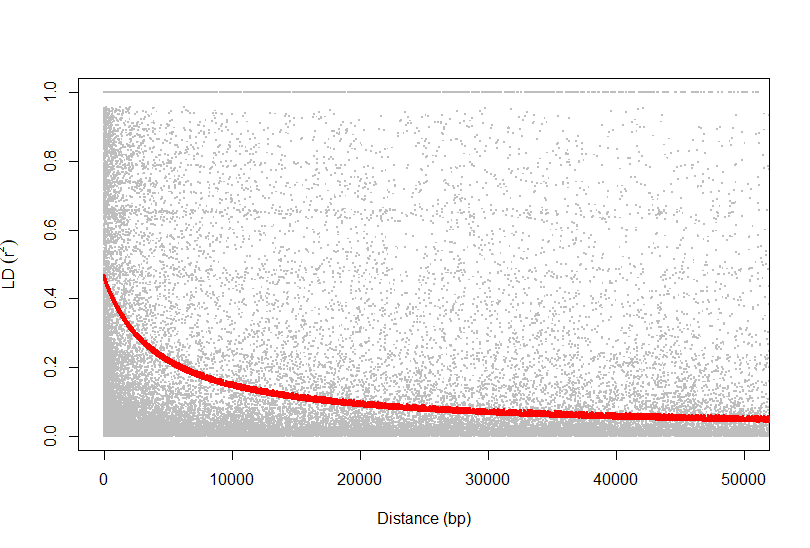


Chromosome 1


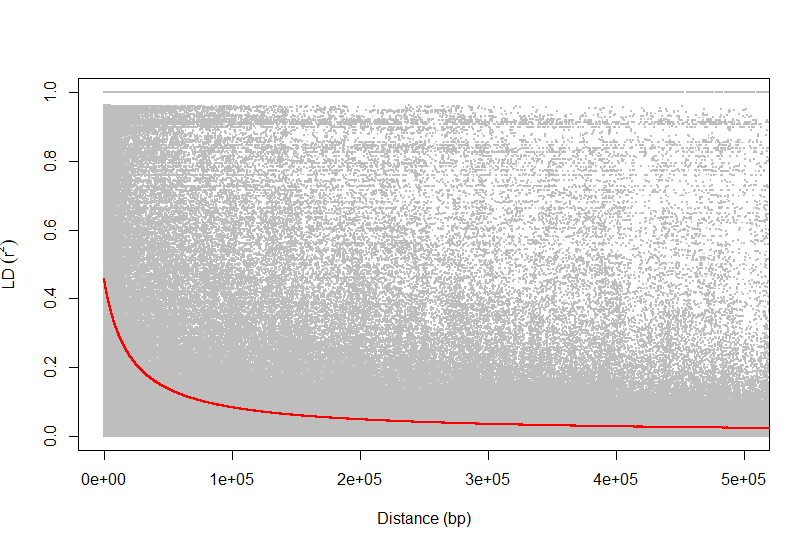


Chromosome 2


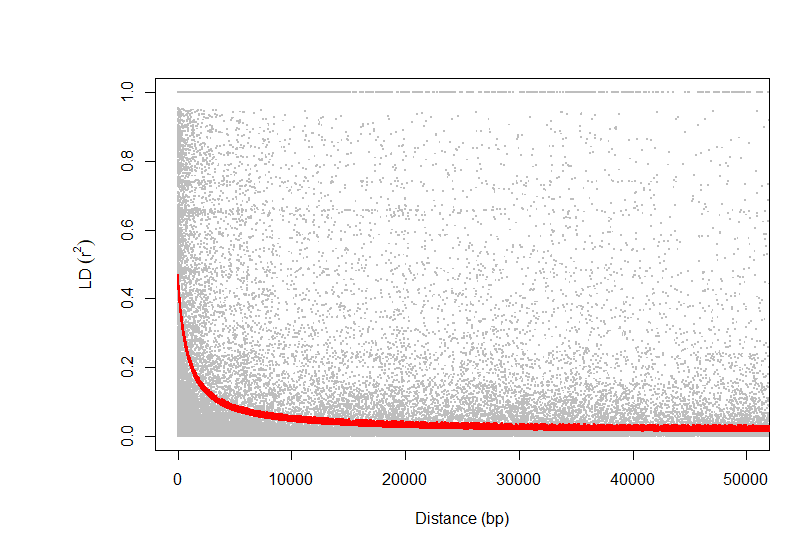


Chromosome 6


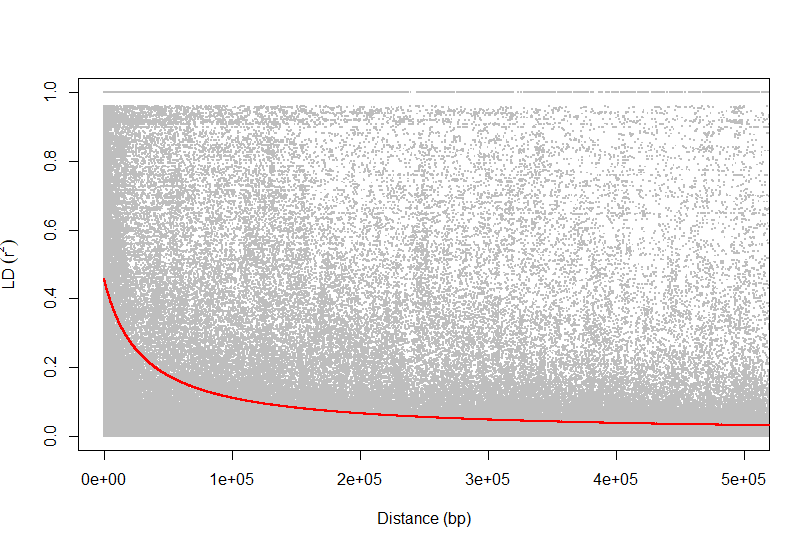


Chromosome 5

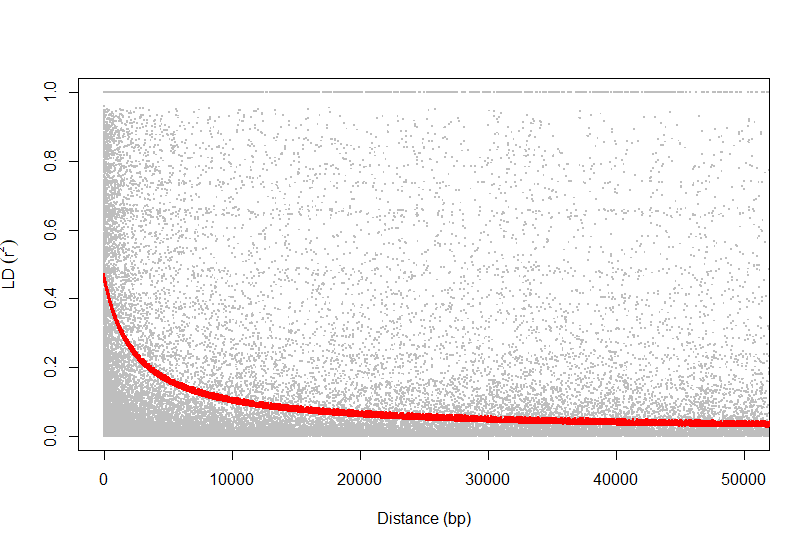


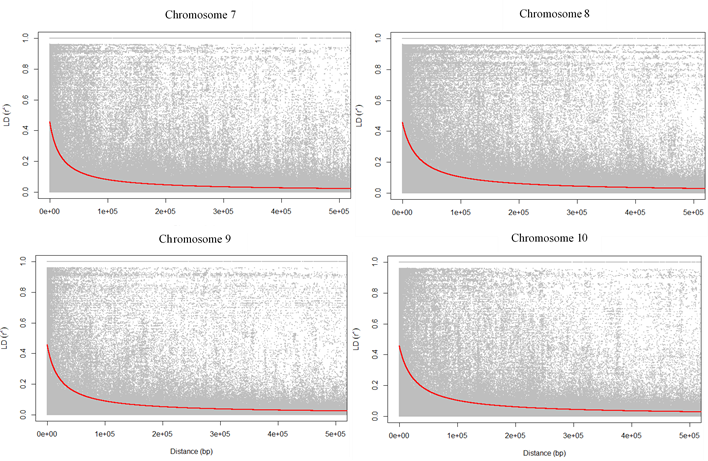


Figure S1. The linkage disequilibrium (LD) decay results for the 10 chromosomes of the maize dataset.

Table S1. The top 25 superior hybrid combinations of the pumpkin dataset identified by Wu et al. (2019) and our study*.* The colored ones are the common hybrids selected by both approaches.

| Our study | Wu *et al.* (2019) |
| --- | --- |
| P026⨂P236 | P026⨂P236 |
| P026⨂P234 | P026⨂P027 |
| P026⨂P027 | P026⨂P235 |
| P026⨂P235 | P026⨂P234 |
| P026⨂P028 | P026⨂P237 |
| P026⨂P237 | P026⨂P028 |
| P234⨂P313 | P227⨂P236 |
| P235⨂P313 | P227⨂P235 |
| P236⨂P313 | P227⨂P234 |
| P028⨂P313 | P026$\boldsymbol{\bigotimes}$P233 |
| P227⨂P236 | P227$\bigotimes$P237 |
| P227⨂P235 | P028⨂P227 |
| P027⨂P313 | P027⨂P227 |
| P227⨂P234 | P026$\boldsymbol{\bigotimes}$P138 |
| P028⨂P227 | P026⨂P253 |
| P236⨂P252 | P026⨂P255 |
| P027⨂P227 | P026⨂P254 |
| P237⨂P313 | P026⨂P302 |
| P007⨂P026 | P007⨂P026 |
| P026⨂P302 | P008$\boldsymbol{\bigotimes}$P026 |
| P227$\boldsymbol{\bigotimes}$P237 | P026⨂P252 |
| P235⨂P252 | P026$\boldsymbol{\bigotimes}$P243 |
| P028⨂P252 | P138$\boldsymbol{\bigotimes}$P241 |
| P236⨂P257 | P026$\boldsymbol{\bigotimes}$P241 |
| P234⨂P252 | P100⨂P236 |

Table S2. The top 10 superior parental lines of the pumpkin dataset identified by Wu et al. (2019) and our study*.* The colored ones are the common parental lines selected by both approaches.

| Our study | Wu et al. (2019) |
| --- | --- |
| P236 | P026 |
| P235 | P236 |
| P027 | P235 |
| P234 | P027 |
| P028 | P234 |
| P237 | P237 |
| P026 | P241 |
| P252 | P324 |
| P007 | P028 |
| P302 | P255 |
